# GSK-3α regulates miRNAs associated with transcriptional and metabolic processes in human cardiomyocytes under hypoxia

**DOI:** 10.1042/BCJ20253208

**Published:** 2025-09-09

**Authors:** Firdos Ahmad, Hezlin Marzook, Musa Idris, Omama I. Dawuod, Megna Srinivas, Asima Karim, Mohamed A. Saleh, Rizwan Qaisar

**Affiliations:** 1 Basic Medical Sciences, College of Medicine, University of Sharjah, Sharjah, 27272, United Arab Emirates; 2 Research Institute of Medical and Health Sciences, University of Sharjah, Sharjah, 27272, United Arab Emirates; 3 Department of Pharmacology and Toxicology, Faculty of Pharmacy, Mansoura University, Mansoura, 35516, Egypt; † Department of Clinical Sciences, College of Medicine, University of Sharjah, Sharjah, 27272, United Arab Emirates

**Keywords:** cardiomyocytes, GSK-3α, hypoxia, ischemia, metabolism, microRNA, Inflammation

## Abstract

Glycogen synthase kinase-3α (GSK-3α) is a multifunctional kinase that plays roles in the pathogenesis of various cardiac diseases, including ischemia and pressure overload and ischemia-reperfusion-induced injury. It regulates key cellular processes such as cardiac cell proliferation, apoptosis, metabolism, and inflammation. However, its role in regulating cardiac microRNAs (miRNAs) remains unknown. To explore the role of GSK-3α in regulating miRNAs, we conducted an unbiased miRNA sequencing analysis in human GSK-3α-overexpressing AC16 cardiomyocytes (GOCs) under hypoxic conditions. Transcriptomic analysis demonstrated numerous differentially expressed miRNAs (DEMs) crucial for transcriptional, inflammatory, and various metabolic processes in the cell. Among 184 DEMs, hsa-miR-3934-5p, hsa-miR-139-5p, and hsa-miR-185-5p were the most up-regulated, while hsa-miR-193b-3p, hsa-miR-181a-2-3p, and hsa-miR-369-3p were the most down-regulated in GOC vs. control cells subjected to hypoxia. Gene ontology (GO) term analysis demonstrated a significant set of genes associated with the terms regulation of transcription, cellular protein modification process, cellular aromatic compound metabolic process, and nucleotide binding in GOC. Kyoto Encyclopedia of Genes and Genomes (KEGG) pathway analysis further revealed enrichment of key pathways including metabolic, cytokine–cytokine receptor interaction, cyclic adenosine monophosphate (cAMP), and mitogen-activated protein kinase (MAPK) signaling pathways in GOC challenged with hypoxia. Collectively, these findings reveal a novel mechanism by which GSK-3α regulates a network of miRNAs in human cardiomyocytes required for critical transcriptional, metabolic, and signaling responses including the MAPK and inflammatory pathways under hypoxic stress. GSK-3α-mediated miRNA dysregulation may contribute to the pathophysiological changes observed in ischemia-induced cardiac injury.

## Introduction

Ischemia-induced cardiac remodeling and heart failure (HF) are a leading cause of death [1]. Upon ischemic attack, hypoxia triggers numerous cellular events including metabolic reprogramming, oxidative stress, and inflammation, ultimately leading to cardiomyocyte dysfunction and death [2]. MicroRNA (miRNA), a 20–22-nucleotide-long non-coding RNA molecule, post-transcriptionally regulates gene expression by repressing or degrading mRNA [3,4]. miRNAs have emerged as critical regulators of cardiac development, function, and disease. They are involved in fine-tuning gene expression networks that govern cardiac metabolism, signaling pathways, and stress responses, making them essential for maintaining cardiac homeostasis [5]. The expression profiles of various miRNAs are altered during myocardial infarction (MI), which in turn affects the expression levels of genes involved in apoptosis, fibrosis, angiogenesis, and inflammation [6].

miRNAs modulate key metabolic processes in the heart, including glucose and fatty acid metabolism, which are crucial for maintaining the energy production in cardiomyocytes. miR-1 and miR-133 have been shown to regulate genes involved in glucose metabolism and mitochondrial function, thereby influencing cardiac energy balance [7]. Dysregulation of these miRNAs can lead to metabolic perturbation, contributing to the pathogenesis of cardiac diseases such as hypertrophy and HF [8]. In addition to their involvement in metabolic imbalance, miRNAs are integral components of major cardiac signaling pathways, including the Phosphoinositide 3-kinase (PI3K)-Akt, MAPK, and transforming growth factor-β (TGF-β) pathways, which are critical for cardiac fibrosis, cell survival, growth, and apoptosis. miR-21 has been implicated in the regulation of the PI3K-Akt pathway, promoting cell survival and protecting against cardiac injury [9]. Similarly, miR-208a regulates the expression of genes involved in cardiac hypertrophy and fibrosis by modulating the TGF-β signaling pathway [10]. These findings highlight the important role of miRNAs in the regulation of signaling networks and cardiac pathogenesis.

Accumulating evidence suggests that protein kinases regulate miRNA expression by modulating various steps in miRNA biogenesis and function [11,12]. MAPK/extracellular signal-regulated kinase (ERK) pathway activation can phosphorylate transcription factors (TFs) like Myc, which, in turn, regulates the transcription of specific miRNAs [13]. Additionally, kinases such as Akt and mTOR can influence the stability and processing of miRNA precursors by phosphorylating the components of the miRNA processing machinery. AKT-mediated phosphorylation of TAR RNA-binding protein (TRBP), a component of the Dicer complex, enhances the miRNA processing efficiency [13].

Glycogen synthase kinase-3α (GSK-3α), a serine/threonine kinase, regulates various cellular processes, including oxidative stress, mitochondrial function, cell growth, differentiation, and apoptosis [14–16]. The role of GSK-3α in ischemia- and pressure-overload-induced pathological processes such as cardiomyocyte proliferation and apoptosis, cardiac fibrosis and inflammation, and ventricular remodeling is well established [17–19]. However, its potential impact on the regulation of cardiac miRNAs in response to hypoxia remains unexplored. The potential relationship between GSK-3α and miRNA regulation may provide new insights into the molecular basis of ischemic heart disease. The primary aim of this present study was to investigate the role, if any, of GSK-3α in modulating miRNA expression in human cardiomyocytes under hypoxic conditions.

We employed unbiased miRNA sequencing to analyze the miRNA expression profile in GSK-3α-overexpressing human cardiomyocytes subjected to hypoxic conditions. We identified, for the first time, that gain-of-GSK-3α function in cardiomyocytes dysregulates numerous miRNAs important for cellular signaling and metabolic processes. Specifically, GSK-3α overexpression led to the induction of a significantly higher number of miRNAs associated with transcription, protein modification process, cellular aromatic compound metabolic process, and nucleotide binding post-hypoxia. The major enriched pathways in GSK-3α-overexpressing cardiomyocytes include the PI3K-Akt and MAPK, cAMP, metabolic, and cytokine–cytokine receptor interaction signaling pathways.

## Results

### GSK-3α regulates unique miRNAs in cardiomyocytes

**Figure 1:**
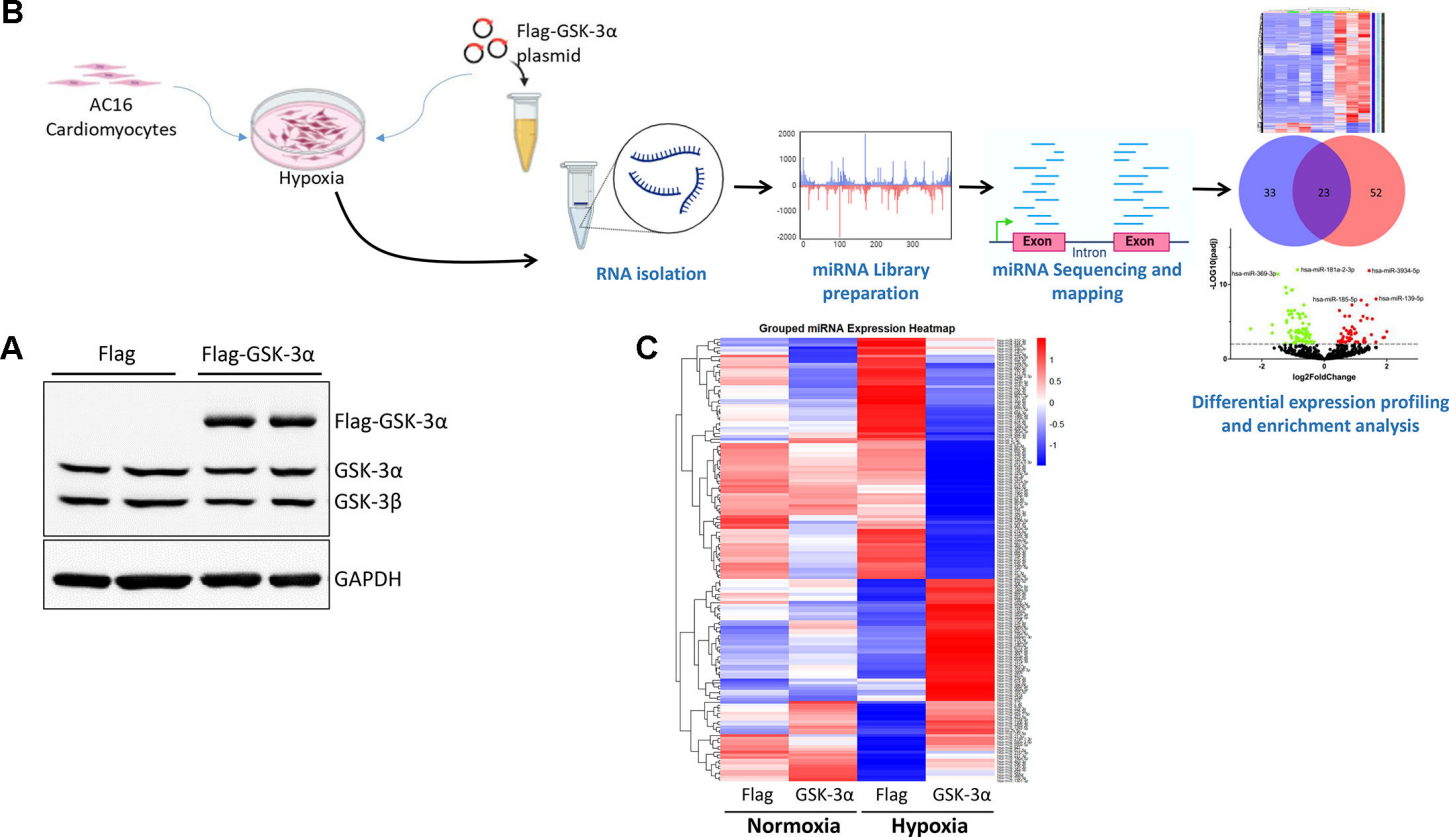
Schematic diagram of miRNA sequencing in GSK-3α-overexpressing cardiomyocytes (**A**) The schematic diagram illustrates the experimental workflow for miRNA sequencing in GSK-3α-overexpressing cardiomyocytes. AC16 cells were transfected with a Flag-GSK-3α or Flag control plasmid and subjected to hypoxia for 24 h. Following hypoxia treatment, RNA was isolated, and mRNA libraries were prepared for sequencing. The sequenced reads were mapped to the reference genome, and differential expression profiling was performed to identify miRNAs regulated by GSK-3α. Enrichment analysis was conducted to explore the biological pathways and processes associated with the differentially expressed miRNA (DEM) under hypoxia. (**B**) Western blotting shows the overexpression of GSK-3α in the AC16 cardiomyocytes transfected with Flag-GSK-3α. (**C**) The grouped heatmap displays the DEM (selected by adjusted *P*-value <0.05 and fold-change >2) in GSK-3α-overexpressing and Flag control cardiomyocytes under hypoxic conditions. Each row represents an miRNA, and each column represents a sample group, with color intensity indicating the level of miRNA expression (red for up-regulated and blue for down-regulated). GSK-3α, glycogen synthase kinase-3α.

To understand the role of GSK-3α in cardiac pathophysiology particularly under ischemic conditions, miRNA sequencing was performed in GSK-3α-overexpressing human cardiomyocytes challenged with hypoxia. Protein overexpression of GSK-3α was first validated (Figure 1A), and then miRNA sequencing and transcriptomic analysis were conducted in the transfected cells (Figure 1B). We performed heat map cluster analysis for miRNA sequencing data to visualize the patterns of miRNA expression profiles in GSK-3α-overexpressing cells under normoxia and hypoxia. The heatmap allowed for the identification of group-specific trends, such as miRNAs that are uniquely regulated in GSK-3α-overexpressing cells under hypoxia. The analysis showed a distinct clustering of miRNA expression patterns between GSK-3α-overexpressing and Flag control cells under both conditions. Under normoxia, the changes in miRNA expression profiles of GSK-3α-overexpressing cells vs. Flag control cells were relatively minor, with differences in the intensity of expression of certain miRNAs. However, under hypoxic conditions, a more pronounced divergence in miRNA expression profile was observed between GSK-3α-overexpressing and Flag control cells (Figure 1C), indicating that GSK-3α overexpression significantly alters miRNA expression, particularly under hypoxic stress.

**Figure 2:**
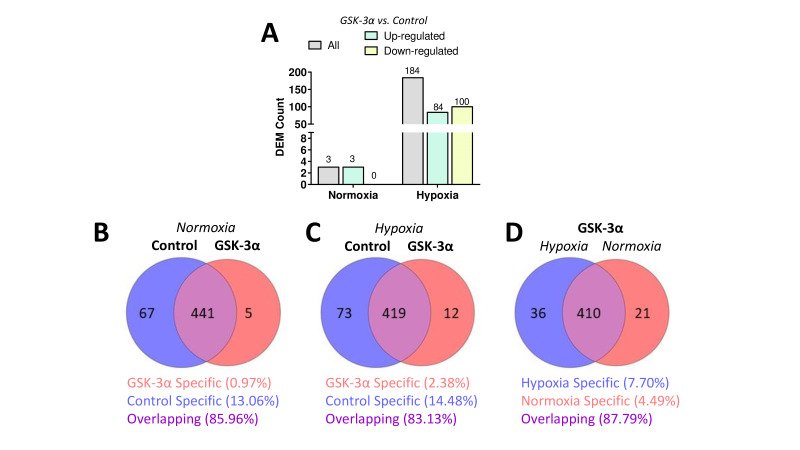
Differential expression and Venn diagrams of miRNAs in GSK-3α-overexpressing cardiomyocytes (**A**) Differentially expressed miRNAs (DEMs) under normoxia and hypoxia. Venn diagrams comparing miRNA expression profiles between GSK-3α-overexpressing and control cells under (**B**) normoxia and (**C**) hypoxia, respectively. (**D**) miRNA expression in GSK-3α-overexpressing cells under hypoxia vs. normoxia. GSK-3α, glycogen synthase kinase-3α.

A relatively smaller number of DEMs were identified in GSK-3α-overexpressing cells compared with Flag control cells subjected to normoxia; however, a significantly increased number of DEMs were identified in GSK-3α-overexpressing cells subjected to hypoxia (Figure 2A). Only three up-regulated miRNAs were identified under normoxia, while 84 miRNAs were found up-regulated and 100 were down-regulated in GSK-3α-overexpressing cells under hypoxia. Venn diagrams provided a detailed comparison of miRNA expression profiles in GSK-3α-overexpressing and Flag control cardiomyocytes under normoxic and hypoxic conditions. A total of five miRNAs were uniquely expressed in GSK-3α-overexpressing cells, while 67 miRNAs were uniquely expressed in Flag control cells under normoxia conditions. The other 441 miRNAs were commonly expressed in both GSK-3α-overexpressing and Flag control cells under normoxia (Figure 2B). The number of uniquely expressed miRNAs increased to 12 in GSK-3α-overexpressing cells, and to 73 in Flag control cells when subjected to hypoxia (Figure 2C). Next, we assessed the uniquely expressed miRNAs in GSK-3α-overexpressing cells under the hypoxic vs. normoxic conditions. While 36 miRNAs were uniquely regulated by hypoxia and 21 by normoxia, 410 miRNAs were consistently expressed in GSK-3α-overexpressing cells under both conditions (Figure 2D). These findings suggest the role of GSK-3α in modulating miRNA expression, particularly under hypoxic stress.

### GSK-3α differentially regulates miRNAs in cardiomyocytes under hypoxia

**Figure 3:**
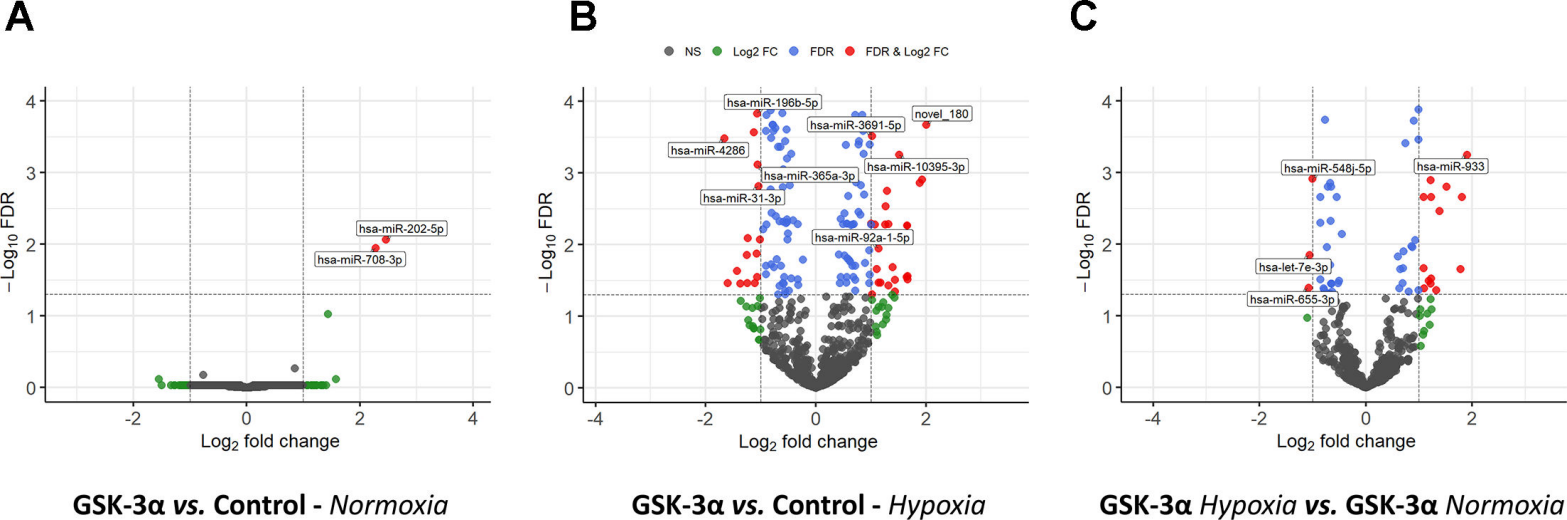
Volcano plots of DEMs in GSK-3α-overexpressing cardiomyocytes Volcano plots show DEMs in GSK-3α-overexpressing vs. control cardiomyocytes under (**A**) normoxia, (**B**) hypoxia, and (**C**) a comparison of GSK-3α-overexpressing cells under hypoxia vs. normoxia. Gray dots show miRNAs with no significant change in expression, red dots represent significantly up-regulated or down-regulated miRNAs. GSK-3α, glycogen synthase kinase-3α.

Further analysis of transcriptomic data through volcano plot construction provided a detailed visualization of DEMs. Under normoxia, most miRNA clusters were observed to be located around the center of the plot, indicating minimal fold changes and no significant differential expression. A few miRNAs showed slight up-regulation or down-regulation, but none reached high statistical significance (e.g. log2FoldChange >1 or <-1 with adjusted *P*<0.05) except for hsa-miR-202–5p and hsa-miR-708–3p, which were significantly up-regulated in GSK-3α-overexpressing cells (Figure 3A **,** Table 1). In contrast, several miRNAs were significantly up-regulated or down-regulated under hypoxia. Notably, miRNAs including hsa-miR-3934–5p, hsa-miR-185–5p, and hsa-miR-139–5p were prominently up-regulated, while several others including hsa-miR-193b-3p, hsa-miR-369–3p, and hsa-miR-181a-2–3p were among the top down-regulated in GSK-3α-overexpressing cells post-hypoxia (Figure 3B, Table 1). The novel miRNA, novel_180, was among the most up-regulated miRNAs, and its predicted secondary structure is provided as Supplementary Figure S1. To identify the DEMs specifically regulated by hypoxia, a comparative analysis was performed between GSK-3α-overexpressing cells challenged with hypoxia and normoxia. The expression profiles of several miRNAs were significantly modulated under hypoxia vs. normoxia. Notably, miRNAs including hsa-miR-210–5p, hsa-miR-1246, and hsa-miR-1260b were among the top up-regulated, while novel_134, hsa-miR-494–3p, hsa-miR-196a-5p, and hsa-miR-181b-5p were among the top down-regulated miRNAs in the GSK-3α-overexpressing cells post-hypoxia vs. normoxia (Figure 3C, Table 1). The master list of DEMs is provided as Supplementary Table S1. These observations reveal that GSK-3α overexpression significantly affects miRNA expression under hypoxic conditions.

**Table 1:** Top differentially regulated miRNAs (based on *P*-adjusted values) in GSK-3α-overexpressing cardiomyocytes.

GSK-3α vs. control - Normoxia							
Up-regulated				Down-regulated – No Significant Genes			
No.	Gene	*P*-adj	Log_2_FoldChange	No.	Gene	*P*-adj	Log_2_FoldChange
1	hsa-miR202–5p	0.0086	2.456				
2	hsa-miR-708–3p	0.0112	2.2788				
3	novel_180	0.0944	1.4356				
**GSK-3α vs. control - Hypoxia**							
**Up-regulated**				**Down-regulated**			
**No.**	**Gene**	***P*** **-adj**	**Log**_**2**_ **FoldChange**	**No.**	**Gene**	***P*** **-adj**	**Log**_**2**_ **FoldChange**
1	hsa-miR-3934–5p	5.83E–15	1.4366	1	hsa-miR–193b-3p	1.08E–23	-1.3366
2	hsa-miR-139–5p	1.10E–10	1.6573	2	hsa-miR-181a-2–3p	1.06E–12	–0.855
3	hsa-miR-185–5p	1.81E–10	1.18	3	hsa-miR–369–3p	4.11E-12	–1.4831
4	hsa-miR–129–5p	9.87E–10	1.3666	4	hsa-miR–15b-5p	2.44E–10	–1.2347
5	hsa-miR–584–5p	1.01E–09	0.8862	5	hsa-miR-199a–3p	5.16E–10	–1.0164
6	hsa-miR–130b-5p	6.55E–09	0.4867	6	hsa-miR–493–5p	5.69E–10	–1.0457
7	hsa-miR–34a-5p	6.34E–08	0.6589	7	hsa-miR–494–3p	1.50E–09	–1.2094
8	hsa-miR–543	6.41E–08	0.8056	8	hsa-miR–98–5p	5.60E–08	–0.6527
9	hsa-miR–1246	7.19E–08	1.241	9	hsa-miR–1271–5p	3.00E–07	–0.9493
10	hsa-miR-3679–5p	1.56E-07	1.3731	10	hsa-miR-339–5p	3.35E-07	-1.1531
11	hsa-miR-1268a	1.94E-07	1.5386	11	hsa-miR-27b-3p	3.66E-07	-1.0513
12	hsa-miR-877–5p	3.36E-07	1.1666	12	hsa-miR-214–5p	8.27E-07	-0.6792
13	hsa-miR-744–5p	2.72E-06	0.8942	13	hsa-miR-3120–3p	8.27E-07	-0.6792
14	hsa-miR-485–5p	4.06E-06	0.6321	14	hsa-miR-329–3p	8.27E-07	-0.8431
15	hsa-miR-2110	5.88E-06	0.9676	15	hsa-miR-199a-5p	9.16E-07	-1.0466
16	hsa-miR-151a-3p	1.09E-05	0.8439	16	hsa-miR-23b-3p	1.08E-06	-1.1238
17	hsa-miR-3909	1.08E-05	0.712	17	hsa-miR-125b-5p	1.09E-06	-0.7837
18	novel_180	1.60E-05	2.0031	18	hsa-miR-758–3p	1.11E-06	-0.7828
19	hsa-miR-3689f	1.72E-05	0.8003	19	hsa-miR-22–3p	2.00E-06	-0.5977
20	hsa-miR-320a-3p	2.11E-05	0.8586	20	hsa-miR-210–3p	2.86E-06	-0.7889
**GSK-3α – Hypoxia vs. normoxia**							
**Up-regulated**				**Down-regulated**			
**No.**	**Gene**	**P-adj**	**Log**_**2**_ **FoldChange**	**No.**	**Gene**	**P-adj**	**Log**_**2**_ **FoldChange**
1	hsa-miR-1260b	1.34E-13	1.3067	1	novel_134	2.86E-09	-2.722
2	hsa-miR-1246	5.80E-08	1.8151	2	hsa-miR-494–3p	4.51E-09	-0.7935
3	hsa-miR-210–5p	6.90E-07	1.595	3	hsa-miR-196a-5p	5.42E-06	-0.897
4	hsa-miR-12136	4.48E-06	1.7711	4	hsa-miR-181b-5p	1.29E-05	-0.7936
5	hsa-miR-33b-3p	1.56E-03	1.515	5	hsa-miR-93–5p	2.69E-05	-0.9117
6	hsa-miR-3934–5p	2.16E-03	1.0845	6	hsa-miR-329–3p	3.78E-05	-1.0299
7	hsa-miR-4677–5p	2.16E-03	1.8084	7	hsa-miR-125a-5p	3.78E-05	-0.9733
8	hsa-miR-412–5p	2.16E-03	1.2244	8	hsa-miR-125b-5p	1.81E-04	-0.7739
9	hsa-miR-3918	3.40E-03	1.3847	9	hsa-miR-942–5p	7.88E-04	-0.9499
10	hsa-miR-365b-5p	8.73E-03	0.9252	10	hsa-miR-548j-5p	1.21E-03	-1.005
				11	hsa-miR-484	1.39E-03	-0.675
				12	hsa-miR-181a-2–3p	1.56E-03	-0.7138
				13	hsa-let-7d-5p	1.56E-03	-0.6541
				14	hsa-miR-485–3p	2.16E-03	-0.8583
				15	hsa-miR-22–3p	2.16E-03	-0.5488
				16	hsa-miR-328–3p	4.67E-03	-0.6653
				17	hsa-miR-369–3p	5.01E-03	-0.8541
				18	hsa-miR-505–3p	7.19E-03	-0.4573

### GSK-3α regulates miRNAs affecting genes associated with transcription and cellular metabolic processes

**Figure 4:**
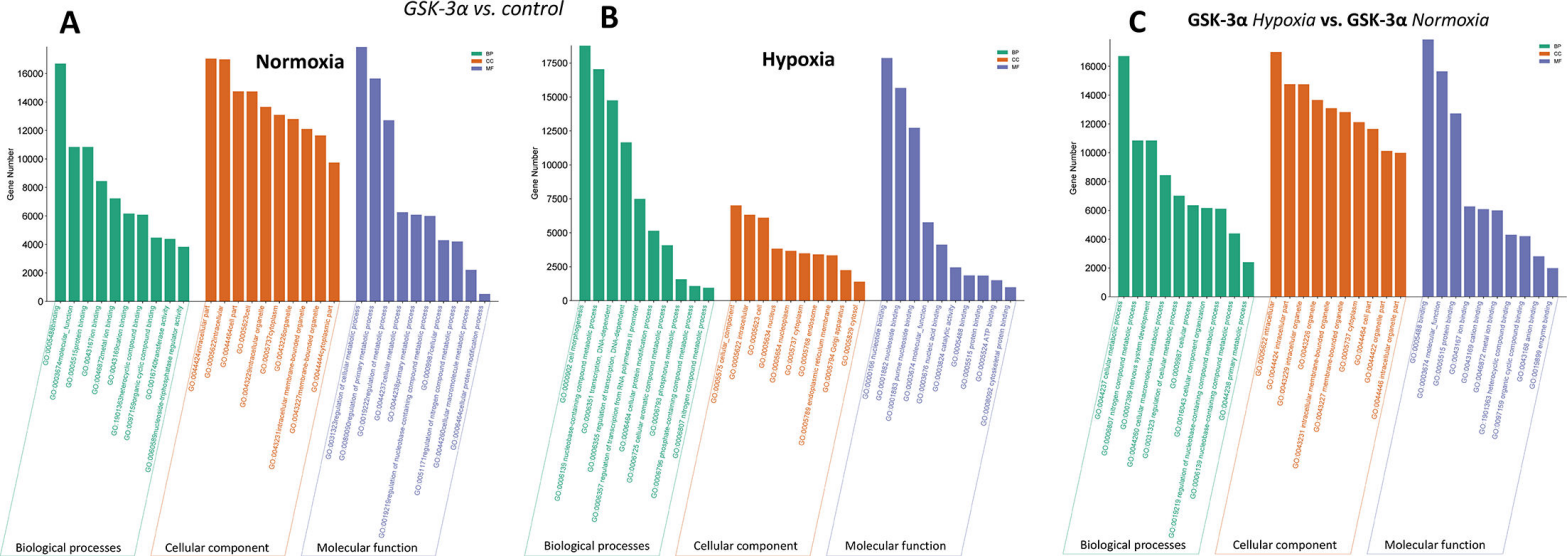
Gene ontology analysis of target genes regulated by DEMs The figures show GO term enrichment analysis of target genes regulated by DEMs in GSK-3α-overexpressing cardiomyocytes under (**A**) normoxia, (**B**) hypoxia, and (**C**) a comparison of GSK-3α-overexpressing cells under hypoxia vs. normoxia. Enrichment analysis highlighting the biological processes, molecular functions, and cellular components significantly enriched in different conditions. GSK-3α, glycogen synthase kinase-3α.

To study the functional activities of dysregulated miRNAs in the biological and cellular processes and related pathways, the target genes were predicted, and their functions were enriched. The gene ontology (GO) study revealed that the enriched target functions in GSK-3α-overexpressing cells were mostly associated with cell metabolism and transcriptional regulation mechanisms. Particularly, the GO terms related to metabolic processes (GO:0006139, GO:0006725, and GO:0006796), transcription (GO:0006351, GO:0006355, and GO:0006357), and nucleic acid binding (GO:0003676) were prominently enriched in overexpressing cells subjected to hypoxia (Figure 4A–C).

### GSK-3α regulates pathways required for cell survival and metabolic adaptation in hypoxia

**Figure 5:**
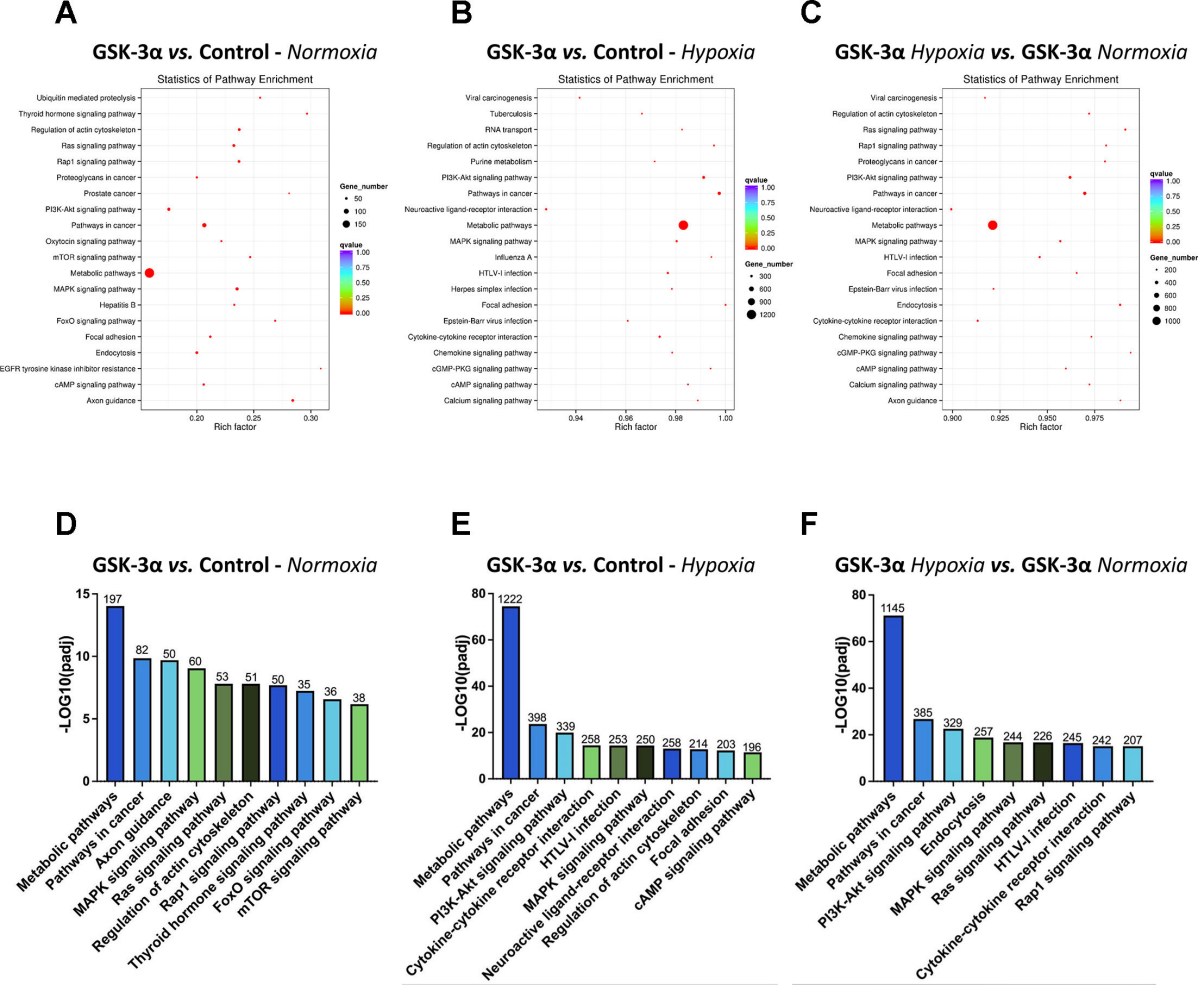
KEGG pathway enrichment analysis of miRNA targets in GSK-3α-overexpressing cardiomyocytes The scatter plot of KEGG pathway enrichment analysis for target genes regulated by DEMs in GSK-3α-overexpressing cardiomyocytes under (**A**) normoxia, (**B**) hypoxia, and (**C**) a comparison of GSK-3α-overexpressing cells under hypoxia vs. normoxia, The x-axis represents the Rich Factor (ratio of differentially expressed genes to total genes in the pathway), and the y-axis represents the −log10 (*P*-value). The bar diagrams show the number of genes enriched in different pathways (based on log10(*P*-adj)) under (**D**) normoxia, (**E**) hypoxia, and (**F**) a comparison of GSK-3α-overexpressing cells under hypoxia vs. normoxia. **GSK-3α**, Glycogen synthase kinase-3α; **cAMP**, Cyclic adenosine monophosphate; **MAPK**, mitogen-activated protein kinase; **PI3K**, Phosphoinositide 3-kinase; **FOXO**, Forkhead box O; **mTOR**, Mammalian target of rapamycin.

KEGG pathway analysis was performed to investigate the potential impact of DEMs on genes associated with different cellular pathways. The analysis revealed that pathways including MAPK, cAMP, PI3K-Akt, metabolic and inflammatory pathways, including cytokine–cytokine receptor interaction and chemokine signaling were most enriched with the highest rich factor in GSK-3α-overexpressing cells challenged with hypoxia (Figure 5A–C). The total number of dysregulated genes associated with metabolic pathways was 197 under normoxia, which was significantly increased to 1222 in cells challenged with hypoxia. Moreover, a total of 60 genes were found associated with PI3K-Akt, 60 with MAPK, and 41 dysregulated genes with cAMP pathways in GSK-3α-overexpressing cardiomyocytes under normoxia (Figure 5D). These gene numbers increased to 339 associated with PI3K-Akt, 250 genes with MAPK, and 196 genes with cAMP pathways in GSK-3α-overexpressing cardiomyocytes subjected to hypoxia (Figure 5E). The number of genes enriched in these pathways remained higher in GSK-3α-overexpressing cells for hypoxia vs. normoxia comparison (Figure 5F), but these numbers were lower than those in the hypoxia group. These observations suggest that GSK-3α overexpression significantly affects the activity of cellular pathways under hypoxia, with a strong influence on pathways related to cell survival, stress responses, inflammation, and metabolism.

### Integrated analysis of CVD Atlas and HMDD

Next, we performed an integrated analysis between identified miRNAs and various cardiovascular diseases (CVDs) based on data from the CVD Atlas and Human microRNA Disease Database (HMDD). The analysis, in the GSK-3α-overexpressing vs. control group under hypoxia, revealed that miRNAs such as hsa-mir-21, hsa-mir-1, hsa-mir-199a, hsa-mir-29b, hsa-mir-23a, and hsa-mir-129 were significantly linked to multiple CVDs, including atherosclerosis, MI, HF, and coronary artery disease, with high association scores (Supplementary Table S2). hsa-mir-21 appeared in 76 different studies relating it to CVDs, followed by hsa-mir-1 46 times and hsa-mir-199a 22 times in relation to different CVDs, demonstrating their broad relevance in cardiovascular pathology. The HMDD data further supported these findings, providing evidence of the role of miRNAs as biomarkers, therapeutic targets, or regulators in CVDs, such as hsa-mir-1 and hsa-mir-199a in various CVDs, including HF, cardiac hypertrophy, and cardiomyopathies, and hsa-mir-21 in HF, cardiomyopathies, and vascular diseases. Additionally, the data highlighted the potential of miRNAs in diagnostic applications, with circulating miRNAs such as hsa-mir-21 and hsa-mir-199a-5p identified as promising biomarkers for disease detection and prognosis. These findings collectively emphasize the critical involvement of miRNAs in CVD pathogenesis and their potential clinical applications in diagnosis and treatment.

### Prediction of upstream transcriptional regulators of miRNAs

**Figure 6:**
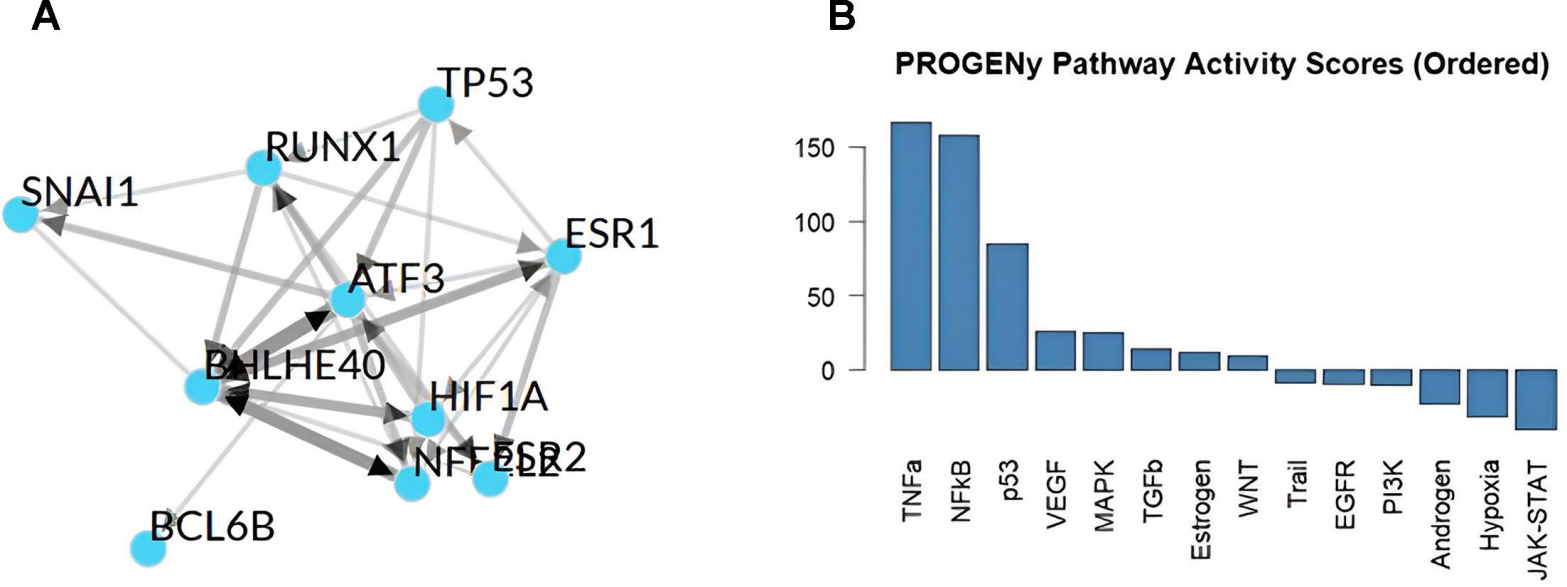
Upstream TFs and the pathways involved. (**A**) A directed gene regulatory network highlighting the key transcriptional interactions among the predicted upstream regulators using ChAE3 platform. Edge thickness reflects interaction strength. (**B**) A bar plot showing PROGENy pathway analysis results comparing active cellular themes in GSK-3α-overexpressing cells under hypoxia vs. Flag. **TP53,** Tumor protein p53; **ESR1,** Estrogen receptor 1; **ESR2,** Estrogen receptor 2; **SNAI1,** Snail family transcriptional repressor 1; **RUNX1,** Runt-related transcription factor 1; **ATF3,** Activating transcription factor 3; **BHLHE40,** Basic helix-loop-helix family member e40; **HIF1A,** Hypoxia-inducible factor 1 alpha subunit; **BCL6B,** B-cell lymphoma 6B; **PROGENy,** Pathway Responsive Genes; **TNFa,** Tumor necrosis factor alpha; **NFkB,** Nuclear factor kappa B; **p53,** Protein 53; **VEGF,** Vascular endothelial growth factor; **MAPK,** Mitogen-activated protein kinase; **TGFβ,** Transforming growth factor beta; **WNT,** Wingless-related integration site; **EGFR,** Epidermal growth factor receptor; **PI3K,** Phosphoinositide 3-kinase; **JAK-STAT,** Janus kinase/signal transducer and activator of transcription.

Our analysis of upstream transcriptional regulators in GSK-3α-overexpressing cardiomyocytes under hypoxia revealed a strong interaction network between key transcriptional factors (TFs) as master regulators of the response to hypoxia in GSK-3α-overexpressing cardiomyocytes. The analysis revealed two groups of TFs: hypoxia-inducible factor-1 alpha (HIF1A), basic helix-loop-helix family member e40 (BHLHE40), and activating transcription factor 3 (ATF3) as master regulators and effectors of hypoxia signaling, while Tumor protein p53 (TP53), Estrogen receptor 1 (ESR1), ESR2, Snail family transcriptional repressor 1 (SNAI1), and Runt-related transcription factor 1 (RUNX1) were found to be involved in GSK-3α -mediated signaling (Figure 6A). Pathway RespOnsive GENes (PROGENy) analysis highlighted the significant involvement of tumour necrosis factor alpha (TNFα) and NF-κB signaling, consistent with a strong pro-inflammatory response in GSK-3α-overexpressing cardiomyocytes under hypoxia (Figure 6B). Moderate p53 activity indicated engagement of apoptotic or stress-related mechanisms. Analysis further revealed the moderate activation of vascular endothelial growth factor (VEGF) and MAPK pathways. Other canonical pathways, including TGFβ, WNT, and PI3K, remained largely inactive. These observations strongly suggest that the primary cellular response in GSK-3α-overexpressing cardiomyocytes under hypoxia is dominated by a robust pro-inflammatory and stress-related program, characterized by a significant activation of the TNFα and NF-κB signaling.

### Validation of top differentially expressed miRNAs

**Figure 7:**
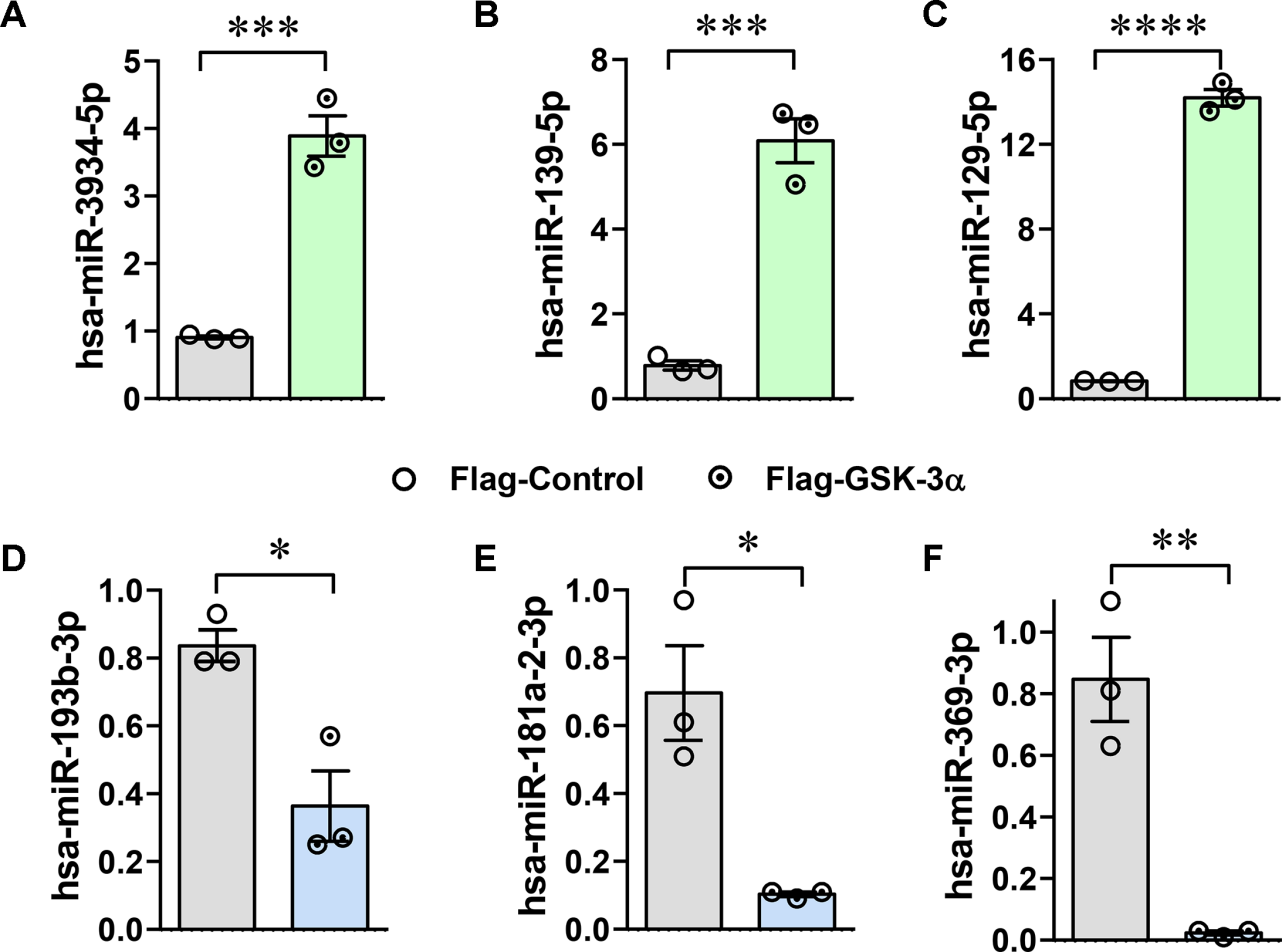
Validation of top DEMs The bar diagrams (**A-F**) show the relative expression levels of selected miRNAs, validated by qRT-PCR, in GSK-3α-overexpressing and control cardiomyocytes under hypoxia. GSK-3α, Glycogen synthase kinase-3α.

The validation through quantitative real-time reverse-transcription PCR (qRT-PCR) confirmed the differential expression of specific miRNAs in GSK-3α-overexpressing cardiomyocytes under hypoxia. As identified through RNA sequencing, the expression of miRNAs, such as hsa-miR-3934–5p, hsa-miR-139–5p, and hsa-miR-129–5p, was up-regulated several-fold (Figure 7A–C), while the expression of hsa-miR-193b-3p, hsa-miR-181a-2–3p, and hsa-miR-369–3p was down-regulated in GSK-3α-overexpressing cells under hypoxia (Figure 7D–F).

## Discussion

The present study provides novel mechanistic insights into the regulation of cardiac miRNAs under hypoxic conditions. Using miRNA sequencing and transcriptomic analysis, we demonstrated that GSK-3α overexpression significantly alters miRNA expression profiles in human AC16 cardiomyocytes subjected to hypoxia. Dysregulation of numerous miRNAs affects pathways that are crucial for cardiac metabolism, stress adaptation, cell survival and death, and inflammatory responses. These findings highlight a previously undefined GSK-3α function in human cardiomyocytes under hypoxia, which mimics the ischemic environment. The observations suggest that GSK-3α-mediated miRNA dysregulation may significantly contribute to the signaling pathways involved in pathological changes during ischemic heart disease.

GSK-3α plays important roles in various pathological processes, including cardiomyocyte hypertrophy, mitochondrial dysfunction, apoptosis, and inflammation [20–22]. Previous studies from our group have shown that GSK-3α activity is reduced in response to ischemia or hypoxia/reoxygenation (H/R) [17,18]. However, even with this reduction in activity, gain-of-function GSK-3α contributes to oxidative stress and promotes cardiomyocyte apoptosis through mechanisms involving mitochondrial dysfunction, inflammation, and metabolic dysregulation under H/R [18]. The current findings demonstrate that GSK-3α overexpression significantly alters the expression of numerous miRNAs in cardiomyocytes subjected to hypoxia. Notably, we identified miR-3934–5p, miR-139–5p, and miR-185–5p along with several other key miRNAs that were significantly up-regulated in GSK-3α-overexpressing cardiomyocytes. While miR-3934–5p has not yet been studied in cardiomyocytes, emerging evidence from different disease models suggests its involvement in cellular apoptosis and secretion of inflammatory cytokines [23,24]. The role of miR-139–5p in cardiac pathogenesis has been well established. A study has demonstrated that the overexpression of miR-139–5p significantly promotes H9c2 cardiomyocyte apoptosis and inhibits H/R-induced cellular autophagy [25]. This is largely due to decreased AMP-activated protein kinase (AMPK), Raptor, and Unc-51 like autophagy-activating kinase 1 (ULK1) activation and increased mTOR activation.

Consistently, miR-139–5p is found up-regulated in patients with acute myocardial infarction (AMI) and, in human umbilical vein endothelial cells (HUVECs) post-hypoxia, attenuates cell viability. In contrast with the ischemia model, miR-139–5p is down-regulated in the cardiac tissue of patients with pressure-overload-induced hypertrophic cardiomyopathy, where its overexpression attenuates cardiomyocyte hypertrophy and expression of HF markers by targeting c-Jun [26]. Given our previous findings that GSK-3α overexpression induces oxidative stress and cardiac cell death post-H/R [18], the observed miR-139–5p up-regulation in GSK-3α-overexpressing cells may adversely contribute to cardiomyocytes' survival under hypoxic conditions.

miR-185–5p, which is up-regulated in GSK-3α-overexpressing cells, has been linked to the promotion of cardiac fibrosis, particularly in patients with sudden cardiac death [27]. Consistent with our finding, miR-185–5p expression has been shown to increase in cardiac fibroblasts in a mouse model of cardiac fibrosis. Importantly, targeting miR-185–5p attenuates pressure-overload-induced cardiac fibrosis in mice. Additionally, the level of circulating miR-185–5p levels are profoundly elevated in patients with arrhythmogenic right ventricular cardiomyopathy [28]. In contrast, its levels are down-regulated in the plasma of patients with AMI, mouse ischemic hearts, and hypoxic HUVECs [29]. The up-regulation of miR-185–5p has been shown to inhibit angiogenesis associated with cardiac systolic dysfunction following MI [29].

In addition to up-regulated miRNAs, several miRNAs, including miR-193b-3p, miR-181a-2–3p, and miR-369–3p, were down-regulated in GSK-3α-overexpressing cells. The differential expression of these miRNAs is likely to have significant functional consequences, as they have been linked to various cellular processes relevant to cardiac function, including cell growth, differentiation, apoptosis, fibrosis, metabolism, and inflammation under ischemic conditions [30–32]. The study observed reduced levels of miR-193b-3p in the rostral ventrolateral medulla (RVLM) of spontaneously hypertensive rats (SHRs) [33]. Overexpression of miR-193b-3p in the RVLM lowered neuronal excitability, sympathetic activity, and blood pressure in SHRs, while its suppression had the opposite effect in normotensive rats. miR-181a-2–3p is linked to inflammation and apoptosis. It targets genes associated with the NF-κB signaling pathway, which plays a critical role in inflammatory responses [34]. Similarly, miR-181a-5p mimetic limits atherosclerosis by reducing NF-κB activation and vascular inflammation [35]. Moreover, miR-369 attenuates hypoxia-induced apoptosis and inflammation. These studies suggest that the down-regulation of these miRNAs in GSK-3α-overexpressing cardiomyocytes under hypoxia may largely contribute to enhanced inflammatory signaling and apoptosis, thereby reducing cell survival under ischemic conditions.

The functional implications of the observed miRNA dysregulation were further supported by GO and KEGG pathway analyses. GO analysis revealed a significant enrichment of genes associated with the regulation of transcription, cellular protein modification process, cellular aromatic compound metabolic process, and nucleotide binding in GSK-3α-overexpressing cells. KEGG pathway analysis further revealed significant enrichment of pathways involved in metabolism, PI3K-Akt signaling, inflammatory, and MAPK signaling pathways. This suggests that GSK-3α-mediated miRNA dysregulation may have a profound effect on cellular metabolism, cell signaling, and inflammatory responses in cardiomyocytes under hypoxic conditions.

The GO terms related to transcription seen in GSK-3α-overexpressing cardiomyocytes highlight the role of miRNAs in modulating the cardiomyocyte transcriptional processes under hypoxia. These effects of miRNAs on transcription can be exerted by targeting TFs, co-factors, or chromatin-modifying enzymes, thereby modulating gene expression in response to stress. Although there is nothing reported on the role of GSK-3α in miRNA regulation, studies have demonstrated that miRNAs can influence cardiomyocyte proliferation and differentiation [36,37]. We previously demonstrated that GSK-3α regulates cardiomyocyte proliferation through regulating cell cyclins and TF [17]. It is shown that miR-1 and miR-133 regulate TFs like serum response factor and myocyte enhancer factor 2, which are important for cardiac gene expression and stress responses [38,39]. There is a possibility that GSK-3α-mediated dysregulation of miRNAs may modulate the expression of such TFs to modulate transcriptional processes.

The MAPK signaling pathway plays a critical role in various cellular processes, including cell proliferation, differentiation, and apoptosis. The activation of the MAPK pathway can lead to both cell survival and cell death depending on the specific context and the duration and intensity of the stimulus [40]. miRNAs have been reported to target molecules of the MAPK pathway and regulate cardiomyocyte proliferation and death [41–43]. A study has shown that fibroblast-specific loss of GSK-3α limits ERK activation and cardiac fibrosis [44]. Consistently, our PROGENy pathway analysis revealed the activation of the MAPK pathway in GSK-3α-overexpression cells post-hypoxia. The observed alterations in miRNA expression may be linked to altered MAPK pathway activation, contributing to the modulation of cell survival and proliferation under hypoxia.

The pathway enrichment analysis further suggests the role of GSK-3α in inflammatory processes. The enrichment of the cytokine–cytokine receptor interaction and chemokine signaling pathways suggests that GSK-3α may modulate the expression of cytokines and chemokines, which play crucial roles in inflammatory responses under ischemic conditions. Moreover, our integrated analysis further identified inflammatory pathway activations in GSK-3α-overexpressing cardiomyocytes under hypoxia. The dominance of TNFα/NF-κB signaling is consistent with previous reports linking GSK-3α to amplified pro-inflammatory responses in cardiac ischemia conditions [45]. This finding is further supported by our previous mRNA transcriptomic studies, which showed that GSK-3α overexpression up-regulates pathways associated with inflammation, including TNF, NF-κB, NOD-like receptor, interleukin-17 (IL-17), and MAPK signaling pathways in cardiomyocytes post-H/R [18]. These results collectively strengthen the evidence for a critical role of GSK-3α in mediating inflammatory processes under cellular stressors.

The metabolic pathway was the most enriched in the GSK-3α-overexpressing cardiomyocytes post-hypoxia. This finding highlights the role of GSK-3α in the regulation of cellular metabolism, particularly under ischemic stress. Hypoxia induces significant metabolic reprogramming in cardiomyocytes to maintain energy production and cellular survival. Metabolic pathways such as glycolysis, fatty acid oxidation, and amino acid metabolism are critical for generating ATP in oxygen-deprived cardiac tissue [8]. Particularly, under hypoxia, cardiomyocytes shift from oxidative phosphorylation to glycolysis as the primary source of ATP production [46]. The enrichment of these pathways indicates that GSK-3α plays a key role in modulating these adaptive responses in cardiomyocytes during ischemic stress. Other metabolic processes that are regulated by GSK-3α include fatty acid uptake and lipid accumulation. A study has shown that GSK-3α promotes fatty acid uptake in cardiomyocytes, eventually leading to lipotoxic cardiomyopathy through phosphorylation of PPARα [47]. Similarly, GSK-3α was reported to promote lipid accumulation and accelerate atherosclerosis in a high-fat-diet mouse model [48]. These reports, consistent with current findings, further demonstrate that GSK-3α is linked to the inflammatory process and endoplasmic reticulum stress for the activation of pro-atherogenic pathways.

While our study identified GSK-3α as a regulator of miRNA expression in hypoxic cardiomyocytes, the precise mechanisms warrant further investigation. The miRNA biogenesis pathway offers several potential control points for GSK-3α-mediated regulation. Notably, the sister kinase GSK-3β isoform has been shown to phosphorylate Drosha at Ser 300/302 to facilitate its correct nuclear localization [49]. Given the high degree of structural similarity between GSK-3 isoforms, particularly in their kinase domains [50,51], it is plausible that GSK-3α may similarly target miRNA processing enzymes, albeit with potentially distinct phosphorylation patterns or regulatory outcomes. This hypothesis is supported by emerging evidence of isoform-specific substrate preferences, where GSK-3α and GSK-3β isoforms show differential affinity for certain primed phosphorylation motifs [52–54]. The unique miRNA signature we identified could therefore result from [1] direct phosphorylation of microprocessor components (Drosha/DGCR8) [2], regulation of maturation machinery (Dicer/TRBP), or [3] indirect effects through TFs. Future studies should employ phosphoproteomics to identify GSK-3α-specific targets in the miRNA biogenesis pathway and to determine how hypoxia alters these phosphorylation events.

In conclusion, the present study provides novel mechanistic insights into the role of GSK-3α in modulating miRNA expression in human cardiomyocytes under hypoxic conditions. Our findings demonstrate that GSK-3α overexpression leads to significant alterations in the miRNA expression profile, affecting pathways crucial for cardiac function, including metabolism, cell signaling (PI3K-Akt and MAPK), and inflammatory responses. These findings suggest that GSK-3α-mediated miRNA dysregulation may significantly contribute to the pathophysiological changes observed in ischemic cardiac injury. Further investigation into the specific roles of these DEMs and their downstream targets will strengthen the molecular mechanisms underlying GSK-3α-mediated cardiac injury and repair.

## Materials and methods

### Cell culture, plasmid transfection, and treatment

AC16 human cardiomyocytes were cultured as previously described [55]. Briefly, cells were grown in Dulbecco’s Modified Eagle’s Medium (DMEM)/Nutrient Mixture F12 (Sigma-Aldrich, #D8437) supplemented with 10% fetal bovine serum (FBS) and 1% penicillin/streptomycin antibiotic cocktail (Sigma-Aldrich, #P4333). The cells were transfected with plasmids as described previously [16]. Briefly, 0.35 × 10^6^ cells were seeded onto six-well plates. At 60% confluence, the cells were serum-starved for 3 h in Opti-MEM (Gibco, #00448). Subsequently, they were transfected with either control Flag plasmid or wildtype human Flag-GSK-3α plasmid using Fugene 6 (Promega, #E2693) at a ratio of 3:1 (DNA: Fugene 6). The transfection mixture was incubated with cells for 4 h in a CO_2_ incubator. After this period, an equal volume of complete DMEM containing 2X of FBS and penicillin-streptomycin was added to the cells, followed by further incubation for 24 h in a CO_2_ incubator.

AC16 cardiomyocytes were exposed to hypoxia in a regulated Whitley H45 hypoxic chamber. Cells were acclimatized to hypoxic conditions in serum-free media and incubated for 3 h in a CO_2_ incubator. Subsequently, cells were transferred to a hypoxic chamber under a controlled atmosphere of 95% N_2_, 5% CO_2_, and 1% O_2_, which was maintained throughout the 24 h of the hypoxic period as described previously [56].

### Western blotting

Cells were collected using scrapers, and lysates were prepared by centrifugation at 14,000 rpm at 4°C for 20 min. The supernatant was transferred to a fresh tube, and the protein was estimated using a colorimetric protein assay (BioRad #5000006). Western blotting was performed as previously described [57]. Briefly, approximately 20 µg of protein was loaded onto a 12% Tris-glycine sodium dodecyl sulfate-polyacrylamide gel and electrophoresed. The gel was then transferred onto nitrocellulose membranes using a semi-dry method on the Bio-Rad Transblot system (Bio-Rad Trans-Blot Turbo). The membrane was blocked with 5% skimmed milk in 1X TBST for 1 h at room temperature. The membrane was incubated with primary antibodies against GSK-3α/β (Cell Signaling Technology #5676S) and Glyceraldehyde 3-phosphate dehydrogenase (GAPDH) (Fitzgerald #10 R-G109a) overnight at 4°C. The membrane was developed with Clarity Western ECL Substrate kit (Bio-Rad #170–5060), and the blot was visualized using a gel documentation system (BioRadChemidoc Touch Imaging System).

### RNA extraction and quantitative real-time PCR

Total RNA was extracted from AC16 cells using the RNeasy kit (Qiagen #74106), and complementary DNA (cDNA) was synthesized following established protocols [57]. In brief, cells were lysed directly using a lysis buffer containing β-mercaptoethanol and RNase inhibitor, followed by ethanol washing. The RNA was then eluted and resuspended in RNase-free water. RNA quality was evaluated by measuring the OD260/OD280 and OD260/OD230 ratios using a NanoDrop spectrophotometer. cDNA synthesis was performed using the iScript™ cDNA Synthesis Kit (BioRad #170–8891).

A set of top dysregulated miRNAs was randomly selected for qRT-PCR to validate the miRNA-seq data. qRT-PCR was performed as described previously [58]. Briefly, qRT-PCR was performed using the SYBR Green PCR master mix with the appropriate primers (S upplementary T able S 3). Calculations were performed using the delta-delta-Ct method, and the results were normalized to U6 small nuclear RNA (snRNA) expression.

### miRNA library construction, quality control, and sequencing

The 3′ and 5′ adaptors were ligated to the 3′ and 5′ ends of the small RNA, respectively. First-strand cDNA was synthesized following primer hybridization, and a double-stranded cDNA library was generated through PCR amplification. After purification and size selection, the libraries were sequenced using Illumina sequencing with SE50. Quality control measures included quantification of the library using Qubit and qRT-PCR, as well as size distribution analysis using a bioanalyzer. Quantified libraries were pooled and sequenced on Illumina platforms based on effective library concentration and the required data volume.

For quality control, raw reads in Fastq format were processed to obtain clean reads by removing reads containing poly-N, 5′ adapter contaminants, and low-quality reads. The Q20, Q30, and GC content of the raw data were also calculated. Clean reads within a specific length range were selected for all downstream analyses. Known miRNA alignment was performed with mapped small RNA tags using miRBase20.0 as a reference. Novel miRNAs were predicted by using miREvo [59] and miRDeep2 [60]. Target genes for these miRNAs were identified using miRanda. miRNA expression levels were quantified as transcripts per million (TPM), followed by normalization.

### Data analysis

The differential expression analysis of miRNAs between the two conditions/groups was performed using the DESeq2 R package (1.8.3). *P*-values were adjusted as described previously [61]. miRNAs with an adjusted *P*-value <0.05 and absolute log_2_FoldChange >1 were considered differentially expressed. Validated miRNA–target interactions were retrieved using the multiMiR package (1.30.0), restricted to experimentally supported entries. Significant miRNAs (FDR<0.05) from the hypoxia comparison were queried. Volcano plots were generated using the EnhancedVolcano package (1.26.0) to visualize log_2_ fold changes and statistical significance. To identify potential biological functions and pathways associated with the target genes of differentially expressed miRNA, GO enrichment analysis was performed. Furthermore, KOBAS software was used to test the statistical enrichment of target gene candidates in the KEGG pathways.

To systematically analyze miRNA-disease associations in CVDs, we integrated data from two specialized databases: the CVD Atlas (focusing on CVD-specific miRNA-gene-pathway networks) and HMDD v4.0 (a comprehensive repository of experimentally validated miRNA-disease interactions). From the CVD Atlas, we extracted miRNA entries with association scores to CVDs. HMDD was queried for miRNAs related to ‘cardio’, ‘heart’, and ‘vascular’.

To further investigate the upstream regulatory mechanisms and pathway-level responses in GSK-3α-overexpressing cells under hypoxia, two complementary approaches were employed. First, validated miRNA target genes (Entrez IDs) were submitted to the ChEA3 platform using the ‘Integrated’ ranking method to identify enriched TFs. The top ten TFs were selected for local network construction. Second, pathway activity was inferred using the PROGENy algorithm (R package 1.30.0), which estimates pathway scores based on the weighted influence of gene-level log fold changes. A named matrix of log_2_FoldChange values was constructed and scaled prior to analysis.

Differences between qRT-PCR data groups were evaluated for significance using an unpaired t-test (GraphPad Prism Software Inc., San Diego, CA, U.S.A.). Data are presented as the mean ± SEM. A *P*-value <0.05 was considered statistically significant.

## Supplementary material

Online supplementary figure 1

Online supplementary table 1

Online supplementary table 2

Online supplementary table 3

## Data Availability

All the data have been included in the manuscript and supplementary files. miRNA sequencing raw data files were deposited in the NCBI database with BioProject ID #PRJNA1231459.

## Open Access

This article has been published open access under our Subscribe to Open programme, made possible through the support of our subscribing institutions, learn more here: https://portlandpress.com/pages/open_access_options_and_prices#conditional

## Abbreviations

AMI: acute myocardial infarction
CVD: cardiovascular disease
DEMs: differentially expressed miRNAs
DMEM: Dulbecco’s Modified Eagle’s Medium
FBS: fetal bovine serum
GO: gene ontology
GOCs: GSK-3α-overexpressing AC16 cardiomyocytes
GSK-3α: glycogen synthase kinase-3α
HF: heart failure
H/R: hypoxia/reoxygenation
HUVECs: human umbilical vein endothelial cells
MI: myocardial infarction
RVLM: rostral ventrolateral medulla
SHRs: spontaneously hypertensive rats
TF: transcription factor
TF: transcriptional factor
TRBP: TAR RNA-binding protein
cDNA: complementary DNA
miRNA: microRNA
