# Supplementary material for: GSK-3α regulates miRNAs associated with transcriptional and metabolic processes in human cardiomyocytes under hypoxia
Source: Biochem J. 2025 Sep 9;482(18):1321–36. doi: 10.1042/BCJ20253208 (PMC12599231; doi:10.1042/BCJ20253208)
Supplement: Online supplementary figure 1 [file bcj-482-18-BCJ20253208-s001.pdf]

Supplementary Figure 1: Predicted secondary structure of the novel miRNA, Novel_180, illustrating the stem-loop formation and other structural features.

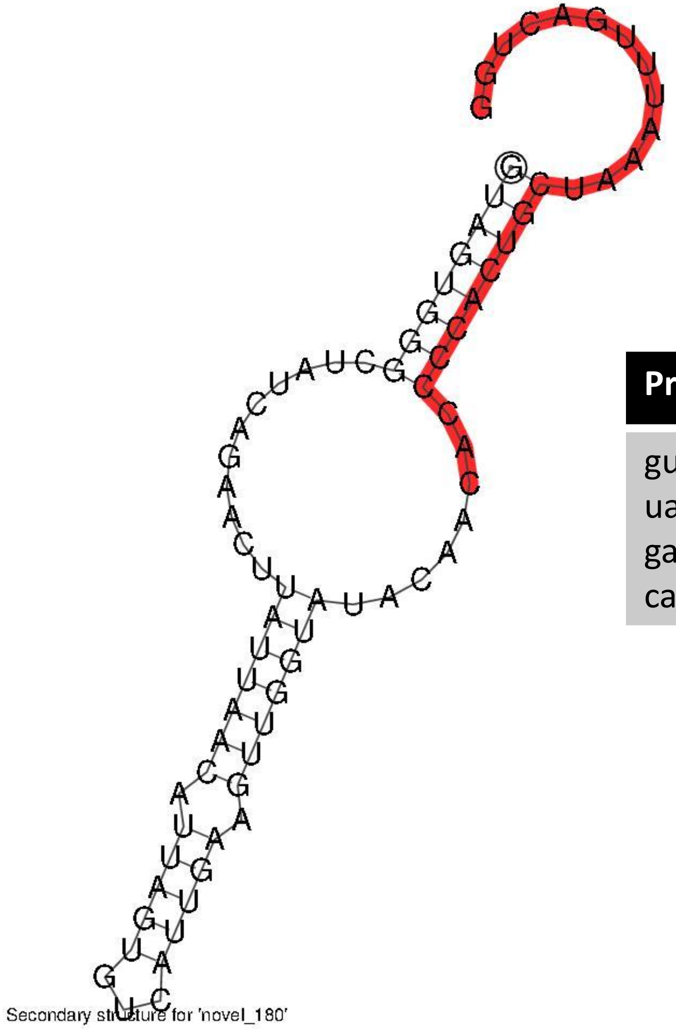

Precursor Sequence	Min Free Energy	Position	Mature Sequence	Mature ID
guagugggcuaucagaacuuauuaacauuagugucauugaaguugguauacaacaccccacugcuaaauuugacugg	-18.8	2:88229574..88229651	caccccacugcuaaauuugacugg	novel_180
