# Supplementary material for: GSK-3α regulates miRNAs associated with transcriptional and metabolic processes in human cardiomyocytes under hypoxia
Source: Biochem J. 2025 Sep 9;482(18):1321–36. doi: 10.1042/BCJ20253208 (PMC12599231; doi:10.1042/BCJ20253208)
Supplement: Online supplementary table 3 [file bcj-482-18-BCJ20253208-s004.docx]

**Suppl. Table 1;** List of primers used in qRT-PCR

| hsa-miR-3934-5p | Forward: GCCAGCTCCTACATCTCAGC |
| --- | --- |
|  | Reverse: AGCCTGACTTGCTAGTGGATTAT |
| hsa-miR-139-5p | Forward: AGTGCACGTGTCTCCAG |
|  | Reverse: GAACATGTCTGCGTATCTC |
| hsa-miR-129-5p | Forward: CTTTTTGCGGTCTGGGCTTG |
|  | Reverse:GAACATGTCTGCGTATCTC |
| hsa-miR-193b-3p | Forward: GGTTTTGAGGGCGAGAT |
|  | Reverse: GAACATGTCTGCGTATCTC |
| hsa-miR-181a-2-3p | Forward: AACATTCAACGCTGTCGGTG |
|  | Reverse: GAACATGTCTGCGTATCTC |
| hsa-miR-369-3p | Forward: TCGACCGTGTTATATTCG |
|  | Reverse: GAACATGTCTGCGTATCTC |
